# Early-onset alopecia universalis successfully treated with baricitinib in a toddler: A case report

**DOI:** 10.1016/j.jdcr.2026.08.014

**Published:** 2026-08-17

**Authors:** Abdulellah Ibrahim Aleissa, Sarah Meteb Alsharif, Adel Alsantli

**Affiliations:** a Department of Dermatology, King Fahad Armed Forces Hospital, Jeddah, Saudi Arabia; b College of Medicine, King Saud bin Abdulaziz University for Health Sciences, Jeddah, Saudi Arabia; c Department of Dermatology, Hair Disorder Unit, King Fahad Armed Forces Hospital, Jeddah, Saudi Arabia

**Keywords:** alopecia universalis, baricitinib, case report, child, Janus kinase inhibitor, pediatric dermatology

## Introduction

Alopecia Areata is a chronic, immune-mediated condition characterized by non-scarring hair loss, with a lifetime risk estimated at approximately 2%.^1^ It encompasses a clinical spectrum ranging from patchy alopecia to more severe forms such as alopecia totalis and alopecia universalis. Among these, alopecia universalis represents the most extensive and least common phenotype, involving complete loss of scalp and body hair.^2^

The disease can occur at any age; however, early-onset cases, particularly in children under 5 years, are associated with increased severity, chronicity, and poorer therapeutic response.^2^^,^^3^ Pediatric alopecia areata poses unique challenges due to its psychological impact, limited treatment options, and concerns regarding long-term safety of systemic therapies.^3^

Clinically, alopecia universalis typically presents with rapid and extensive hair loss. Nail changes, such as pitting, are frequently observed and are associated with more severe disease and a worse prognosis. Although several triggers, including infections and stress, have been implicated, many cases arise without identifiable precipitating factors.^3^ In many cases, no clear precipitating factor is identified, although autoimmune and environmental influences are thought to contribute to disease onset.^1^

Traditional treatment modalities for alopecia areata include topical and intralesional corticosteroids, systemic corticosteroids, and immunosuppressive agents. In recent years, JAK inhibitors such as baricitinib have emerged as promising therapeutic agents. Clinical trials have demonstrated significant hair regrowth in patients with severe alopecia areata treated with baricitinib, highlighting its role as a targeted and effective therapy.^4^ Despite these advances, data regarding their use in young children remain limited, and their application in pediatric alopecia universalis is largely based on case reports and small studies.

Baricitinib, a selective JAK1/2 inhibitor, is Food and Drug Administration-approved (2022) as the first systemic treatment for adults with severe alopecia areata. It is also approved in Europe and USA for moderate-to-severe atopic dermatitis in patients ≥2 years of age and is authorized for hospitalized children with severe COVID-19.^5^ Similarly, tofacitinib (a JAK1/3 inhibitor) is Food and Drug Administration-approved in the US for polyarticular juvenile idiopathic arthritis (including juvenile psoriatic arthritis) in patients ≥2 years old.^6^

In this context, we present a case of early-onset alopecia universalis in a 2-year-old child, successfully treated with baricitinib, highlighting its potential as an effective and safe therapeutic option in this challenging population.

## Case presentation

A 2-year-old girl with a known history of infantile hemangioma on propranolol presented to the dermatology clinic with a sudden onset of diffuse hair loss involving the entire scalp. According to the parents, the hair loss began approximately 15 days prior to presentation and occurred abruptly. The parents reported recent use of an over-the-counter anti-dandruff shampoo containing zinc pyrithione, preceding symptom onset, although there was no preceding history of infection, febrile illness, recent vaccination, or new medication use. There was no personal or family history of atopy, autoimmune disease, or similar hair disorders.

Initial examination revealed complete non-scarring alopecia of the entire scalp, with total loss of eyebrows, eyelashes, and body hair, consistent with alopecia universalis (Fig 1). Nail examination demonstrated pitting, suggestive of nail involvement commonly associated with alopecia areata spectrum disorders. Based on the clinical findings, the patient was diagnosed with alopecia universalis.

**Fig 1:**
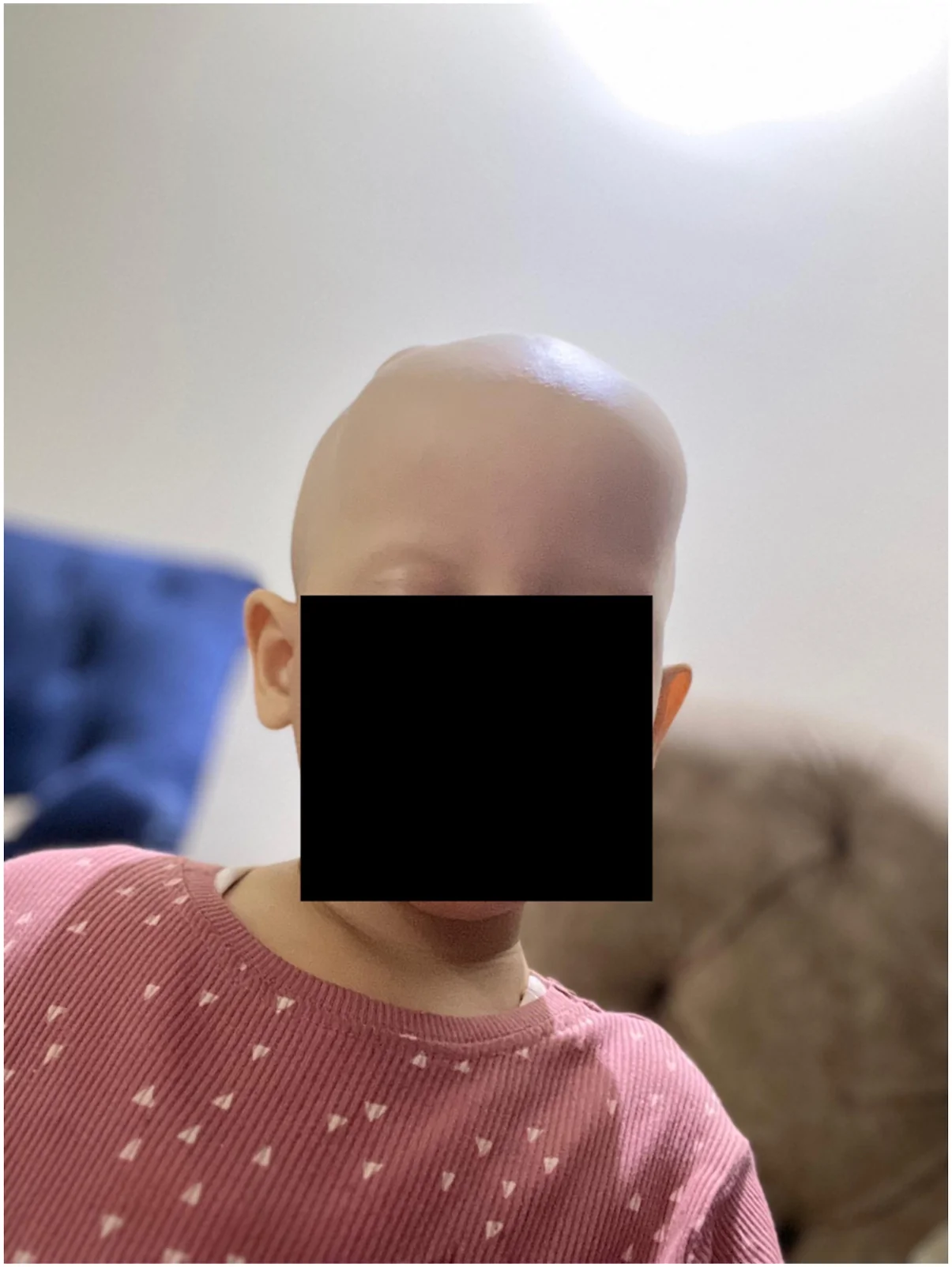
Diffuse nonscarring alopecia involving the scalp and eyebrows at presentation, consistent with alopecia universalis.

Diagnosis was based on clinical presentation. Dermoscopy showed characteristic exclamation mark hairs and yellow dots in affected areas. Fungal culture and potassium hydroxide preparation were negative, ruling out tinea capitis. Scalp biopsy was not performed given the classic clinical features, acute onset, nail involvement, and absence of scarring or inflammation suggestive of other differentials.

Initial topical therapy with clobetasol cream was started; however, only minimal improvement was observed. Given the extensive disease, systemic therapy with baricitinib 2 mg daily was initiated, and the patient was monitored regularly with laboratory investigations. Laboratory monitoring included complete blood count, liver and kidney function tests, lipid profile, and inflammatory markers at baseline, monthly for the first 3 months, and every 3 months thereafter. No significant abnormalities or adverse events were observed throughout the treatment course.

After 6 months of treatment, significant clinical improvement was observed. The patient demonstrated complete regrowth of the eyebrows, along with early regrowing hair in the temporal and occipital scalp regions. Continued treatment led to progressive improvement with regrowth of scalp hair, eyebrows, and eyelashes.

At 4 years of age, the patient achieved complete hair regrowth, consistent with full remission of alopecia universalis (Fig 2). In view of the sustained response, the treatment regimen was tapered to baricitinib 2 mg every other day, which has been maintained for the past 4 months with continued disease control and no relapse. Ongoing follow-up includes clinical assessments every 3 months with plans for gradual further dose reduction or discontinuation if remission persists, alongside long-term surveillance for potential relapse or late adverse effects.

**Fig 2:**
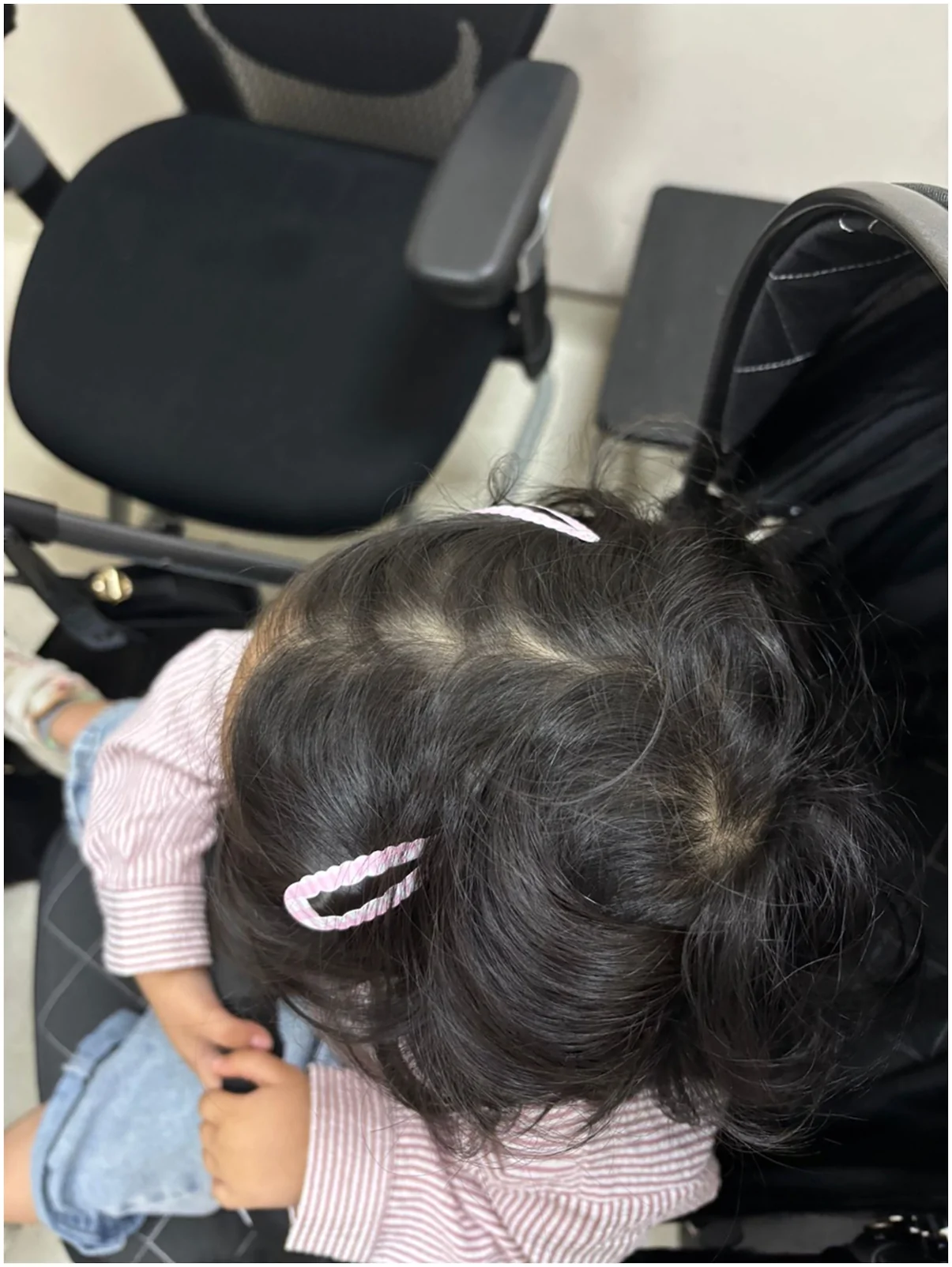
Complete hair regrowth following treatment with baricitinib, demonstrating full remission.

## Discussion

Alopecia Universalis represents the most extensive phenotype within the alopecia areata spectrum, characterized by complete loss of scalp and body hair. It is a rare condition, particularly in the pediatric population, with alopecia areata overall affecting approximately 0.1% to 0.2% of the general population, while alopecia universalis accounts for a much smaller subset of cases.^7^ Pediatric onset, especially before the age of 5 years, is associated with a more severe disease course and poorer prognosis, making our case clinically significant given the early onset at 2 years of age.^3^

The pathogenesis of alopecia universalis is primarily autoimmune, involving T-cell–mediated destruction of hair follicles, particularly through interferon-γ–driven pathways and Janus kinase (JAK)-signal transducer and activator of transcription signaling.^8^ This mechanistic understanding has led to the emergence of JAK inhibitors as targeted therapies. In our patient, the presence of nail pitting further supports the autoimmune etiology, as nail involvement is frequently associated with more severe forms of alopecia areata and is considered a poor prognostic indicator.^9^

Clinically, alopecia universalis typically presents with rapid and diffuse hair loss, which aligns with the abrupt onset described in our case. While triggers such as infections, stress, or medications have been implicated, many cases remain idiopathic. Interestingly, the temporal association with the use of a commercial shampoo in our patient may represent a coincidental finding rather than a causative factor, as there is limited evidence supporting topical products as triggers for autoimmune alopecia.^10^ The absence of preceding illness, vaccination, or drug exposure further supports a spontaneous autoimmune onset.

Management of alopecia universalis remains challenging, particularly in children. Conventional therapies such as topical corticosteroids often yield limited results in extensive disease, particularly in very young children. Systemic immunosuppressants are used but carry risk and variable efficacy.^3^

The introduction of JAK inhibitors has revolutionized the treatment landscape. Baricitinib, an oral JAK1/JAK2 inhibitor, has demonstrated significant efficacy in promoting hair regrowth in severe alopecia areata. Two phase 3 clinical trials showed baricitinib to be superior to placebo in improving scalp hair coverage in adults with severe disease.^4^ Although long-term pediatric data are limited, baricitinib’s mechanism supports its use in severe autoimmune alopecia areata.

Our case is notable for several reasons. First, the patient, who presented at 2 years of age with alopecia universalis, demonstrated an excellent and sustained therapeutic response to baricitinib, achieving progressive and ultimately complete regrowth of scalp hair, eyebrows, and eyelashes over a 2-year treatment period. This outcome contrasts with the typically poor prognosis associated with early-onset alopecia universalis. Second, the patient has maintained full remission on a tapered maintenance dose of baricitinib 2 mg every other day for the past 4 months, with no relapse observed. Importantly, throughout the entire treatment duration, from initiation at age 2 years to the current age of 4 years, the patient has experienced no reported adverse effects, highlighting the potential for safe long-term use in this very young age group with careful monitoring.

Given the off-label use of baricitinib in this 2-year-old patient, a detailed risk-benefit discussion was held with the parents, including potential adverse effects. Written informed consent was obtained for treatment and publication of photographs. The decision was justified by the severe, rapidly progressive nature of the disease, failure of initial topical therapy, poor prognosis of early-onset alopecia universalis, and emerging supportive literature on JAK inhibitors in young children. Close multidisciplinary monitoring was implemented.

The importance of this case lies in its contribution to the limited body of evidence supporting the use of baricitinib in very young children with alopecia universalis. It underscores the potential for early intervention with targeted therapy to alter disease trajectory and achieve complete remission. Furthermore, it highlights the need for larger studies and long-term follow-up data to establish safety, optimal dosing, and duration of therapy in pediatric populations.

## Conclusion

This case highlights the potential of baricitinib as an effective treatment for severe early-onset alopecia universalis. Despite traditionally poor prognostic indicators, complete and sustained hair regrowth was achieved without adverse effects. The findings support the growing role of JAK inhibitors in pediatric dermatology. Further research is required to establish long-term safety and standardized treatment protocols in young children.

## Conflicts of interest

None disclosed.
